# Selection of key sequence-based features for prediction of essential genes in 31 diverse bacterial species

**DOI:** 10.1371/journal.pone.0174638

**Published:** 2017-03-30

**Authors:** Xiao Liu, Bao-Jin Wang, Luo Xu, Hong-Ling Tang, Guo-Qing Xu

**Affiliations:** 1 College of Communication Engineering, Chongqing University, Chongqing, China; 2 Key Laboratory of Chongqing for Bio-perception and Intelligent Information Processing, Chongqing, China; 3 Chongqing City Management College, Chongqing, China; Instituto Nacional de Medicina Genomica, MEXICO

## Abstract

Genes that are indispensable for survival are essential genes. Many features have been proposed for computational prediction of essential genes. In this paper, the least absolute shrinkage and selection operator method was used to screen key sequence-based features related to gene essentiality. To assess the effects, the selected features were used to predict the essential genes from 31 bacterial species based on a support vector machine classifier. For all 31 bacterial objects (21 Gram-negative objects and ten Gram-positive objects), the features in the three datasets were reduced from 57, 59, and 58, to 40, 37, and 38, respectively, without loss of prediction accuracy. Results showed that some features were redundant for gene essentiality, so could be eliminated from future analyses. The selected features contained more complex (or key) biological information for gene essentiality, and could be of use in related research projects, such as gene prediction, synthetic biology, and drug design.

## Introduction

Essential genes are absolutely required for the survival of an organism and are therefore considered the foundation of life [1, 2]. They are useful for biological and biomedical studies, including origin of life, evolution, and drug design studies [1–4]. Two approaches are used to determine essential genes: experimental and computational methods [5]. Experimental methods are time consuming and expensive, and different experimental methods may yield different results [6]. Therefore, computational prediction methods offer a good alternative.

Many computational methods, especially machine learning-based methods, have been proposed for prediction of essential genes [5]. A large range of features that describe the gene essentiality have been adopted for increasing prediction accuracy. Increasing the number of features (i.e., the dimension of feature space) results in a sharp increase in computational complexity and cost. Thus, feature selection is used to aid prediction of essential genes. Saha and Heber selected 13 features based on a modified simulated annealing algorithm, and used them with weighted k-nearest neighbor and support vector machine (SVM) algorithms to classify the essential genes of *Saccharomyces cerevisiae* [7]. Seringhaus *et al*. identified 14 genomic sequence features based on the correlation coefficient, and analyzed the essential genes of both *S*. *cerevisiae* and *Saccharomyces mikatae* [8]. Gustafson *et al*. collected and ranked numerous genomic, protein, and experimental features, and then constructed a classifier for essential gene prediction for *S*. *cerevisiae* and *Escherichia coli* [9]. Hwang *et al*. proposed a method based on genetic algorithms to predict essential genes of *S*. *cerevisiae*, with a backward search-based wrapper for feature selection amongst 31 features [10]. Plaimas *et al*. used a broad variety of metabolic network and sequence features, and trained 100 SVM classifiers to identify genes in *Salmonella typhimurium*. Using their prediction results and an experimental knockout screen, the authors defined 35 enzymes as drug targets [11]. Deng *et al*. presented a machine learning-based integrative approach to predict the essential genes of four bacterial species: *E*. *coli*, *Pseudomonas aeruginosa* PAO1, *Acinetobacter baylyi* ADP1, and *Bacillus subtilis*. Using Bayesian analysis, they ranked all features according to the coverage length of log-odds ratios, and selected 13 features for prediction [12].

Although many features have been proposed and preliminarily selected in these studies, no overall analysis of the widely used features for gene essentiality has been performed. It is important to understand the relationship between the features and gene essentiality, whether all the features are critical to gene essentiality, and which features are key. Moreover, the sample size (analyzed objects) is very limited in existing studies, which affects the generalization ability of the study results (Generalization ability is the ability of a learning machine to perform accurately on new, unseen objects/tasks after having experienced a known, well-studied data set for learning).

Feature selection is a key process in machine learning. The accuracy and generalization capability of classifiers are directly affected by the results of feature selection. The least absolute shrinkage and selection operator (Lasso) is a typical regularized feature selection technique that provides sparsity-inducing estimation of regression coefficients by adding l-1 penalty functions to the traditional least squares regression analysis [13,14]. Lasso has been widely used in fields such as cancer classification and protein inference [15,16].

In this paper, Lasso was used to screen the key feature subset from the most common sequence-based features of gene essentiality, with the selected feature subset assessed by SVM classifiers using 31 bacterial species.

## Materials and methods

### Data source

All the thirty-one bacterial species provided in the DEG11.1 were chosen for examination in this study [17]. Of these, 21 were Gram-negative and ten were Gram-positive (Table 1). The information on the essential/non-essential genes from the bacterial species was obtained from DEG, and the corresponding genome sequences were downloaded from the NCBI GenBank database (ftp://ftp.ncbi.nlm.nih.gov/genomes/Bacteria/). RNA genes, pseudogenes, and other non-coding genes were filtered out, with only protein-coding genes retained. Genes annotated as essential in DEG that matched the corresponding gene from NCBI were marked as positive, while the mismatched genes were marked as negative. Detailed information on these genes is listed in Table 1 and S1 File.

**Table 1:** Information on the 31 bacterial species.

ID	Organism	Abbr.	NCBI Accession ID	Gram	Essential Gene Number	Sample Number
1	*Acinetobacter baylyi* ADP1	ABA	NC_005966	-	498	3307
2	*Bacillus subtilis* 168	BSU	NC_000964	+	271	4175
3	*Bacteroides fragilis* 638R	BFR	NC_016776	-	547	4290
4	*Bacteroides thetaiotaomicron* VPI-5482	BTH	NC_004663	-	325	4778
5	*Burkholderia pseudomallei* K96243	BPS	NC_006350/006351	-	505	5721
6	*Burkholderia thailandensis* E264	BUT	NC_007650/007651	-	403	5631
7	*Campylobacter jejuni subsp*. *jejuni* NCTC 11168 = ATCC 700819	CJE	NC_002163	-	222	1572
8	*Caulobacter crescentus*	CCR	NC_011916	-	401	3182
9	*Escherichia coli* MG1655II	ECO	NC_000913	-	296	4140
10	*Francisella novicida* U112	FNO	NC_008601	-	390	1719
11	*Haemophilus influenzae* Rd KW20	HIN	NC_000907	-	625	1602
12	*Helicobacter pylori* 26695	HPY	NC_000915	-	305	1457
13	*Mycobacterium tuberculosis* H37Rv	MTU	NC_000962	+	599	3872
14	*Mycoplasma genitalium* G37	MGE	NC_000908	+	378	475
15	*Mycoplasma pulmonis* UAB CTIP	MPU	NC_002771	+	309	782
16	*Porphyromonas gingivalis* ATCC 33277	PGI	NC_010729	-	463	2089
17	*Pseudomonas aeruginosa* PAO1	PAE	NC_002516	-	116	5476
18	*Pseudomonas aeruginosa* UCBPP-PA14	PAU	NC_008463	-	335	5892
19	*Salmonella enterica serovar Typhi*	STY	NC_004631	-	347	4195
20	*Salmonella enterica serovar Typhimurium* SL1344	STS	NC_016810	-	353	4446
21	*Salmonella enterica subsp*. *enterica serovar Typhimurium str*. 14028S	SET	NC_016856	-	104	5233
22	*Salmonella typhimurium* LT2	SLT	NC_003197	-	228	4363
23	*Shewanella oneidensis* MR-1	SON	NC_004347	-	402	4065
24	*Sphingomonas wittichii* RW1	SWI	NC_009511	-	535	4850
25	*Staphylococcus aureus* N315	SAU	NC_002745	+	302	2582
26	*Staphylococcus aureus* NCTC 8325	SAN	NC_007795	+	345	2751
27	*Streptococcus pneumoniae*	SPN	NC_003098	+	129	1793
28	*Streptococcus pyogenes* MGAS5448	SPM	NC_007297	+	227	1865
29	*Streptococcus pyogenes* NZ131	SPZ	NC_011375	+	241	1700
30	*Streptococcus sanguinis*	SSA	NC_009009	+	218	2270
31	*Vibrio cholerae* N16961	VCH	NC_002505/002506	-	580	3351

### Subset dataset

Sequence-based features that are widely used in existing prediction models were collated, as shown in Table 2. Because of the differences in cell structure between Gram-negative and Gram-positive bacteria, these two groups have different subcellular localization characteristics. Pre-computed subcellular localization was parsed and added to our dataset. According to our previous work, the Hurst exponent, which represents the long-range correction in a sequence of essential and nonessential genes, is related to the gene essentiality [18,19]. Therefore, despite not being considered in related studies, the Hurst exponent was chosen as a feature in the current work.

**Table 2:** Original features and results of selected features.

	Abbreviations	Description	Selection Results			Tool
			GN	GP	Full	
Intrinsic feature	Gene size	Length of genes		*	*	
	strand	Negative or positive strand		*		
	protein size	Length of amino acids			*	
Codon bias	T3s	Silent base compositions about T	*	*	*	CodonW [20]
	C3s	Silent base compositions about C	*		*	
	A3s	Silent base compositions about A		*	*	
	G3s	Silent base compositions about G	*	*	*	
	CAI	Codon Adaptation Index	*	*	*	
	CBI	Codon Bias Index		*		
	Fop	Frequency of Optimal codons				
	Nc	The effective number of codons		*	*	
	GC3s	G+C content 3rd position of synonymous codons				
	GC	G+C content of the gene	*	*		
	L_sym	Length of system amino acids	*			
	Gravy	Hydropathicity of protein		*	*	
	Aromo	The frequency of aromatic amino acids				
Amino acid usage	Amino acid	A, R, D, C, Q, H, I, N, L, K, M, F, P, S, T, W, Y, V	*			
	Amino acid	R, D, C, E, H, L, G, N, K, F, P, S, T, M, V		*		
	Amino acid	A, R, C, Q, D, H, I, G, N, L, K, M, F, P, S, T, W, V, Y			*	
	Rare_aa_ratio	The frequencies of rare amino acids		*		
	Close_aa_ratio	The number of codons that one third-base mutationis removed from a stop codon				
Physio- chemical Properties	M_weight	Molecular weight				Pepstats [21]
	I_Point	Isoelectric Point		*	*	
	Tiny	Number of mole of the amino acids (A+C+G+S+T)	*	*	*	
	Small	Number of mole of the amino acids (A+B+C+D+G+N+P+S+T+V)				
	Aliphatic	Number of mole of the amino acids (A+I+L+V)	*	*		
	Aromatic	Number of mole of the amino acids (F+H+W+Y)	*	*	*	
	Non-polar	Number of mole of the amino acids (A+C+F+G+I+L+M+P+V+W+Y)	*		*	
	Polar	Number of mole of the amino acids (D+E+H+K+N+Q+R+S+T+Z)	*		*	
	Charged	Number of mole of the amino acids (B+D+E+H+K+R+Z)	*	*		
	Basic	Number of mole of the amino acids (H+K+R)	*		*	
	Acidic	Number of mole of the amino acids (B+D+E+Z)		*	*	
Transmembrane helices	ExpAA	The number of transmembrane amino acids		*		TMHMM3 [22]
	First60	The number of transmembrane amino acids in first 60	*	*	*	
	PredHel	The final prediction of transmembrane helices				
Subcellular localization	Cytom	Cytoplasmic Membrane Score				PSORTb v3.0 [23]
	Extra	Extracellular Score	*	*	*	
	OuterM	Outer Membrane Score				
	Peri	Periplasmic Score	*			
	Cyto	Cytoplasmic Score	*	*	*	
	Cellw	Cell wall Score		*		
	Loc_s	Final Score	*	*	*	
Hurst exponent	Hurst	The Hurst exponent	*		*	R package [24]
Total features (dimension)			37	38	40	

* indicates a selected feature. If a feature was selected from two or three of the sets (GN, GP, Full), then it should be considered significantly associated with essentiality.

Three datasets were constructed: a Gram-negative (GN) dataset, a Gram-positive (GP) dataset, and the Full dataset. The original dimensions of features of the GN and GP datasets were 59 and 58, respectively. The Full dataset contained 57 features, as common subcellular localization features were extracted from GN and GN bacteria. (Note: Each amino acid was treated as one single feature. There were 20 features contained in Amino acid usage.)

### Workflow of data processing

**Fig 1:**
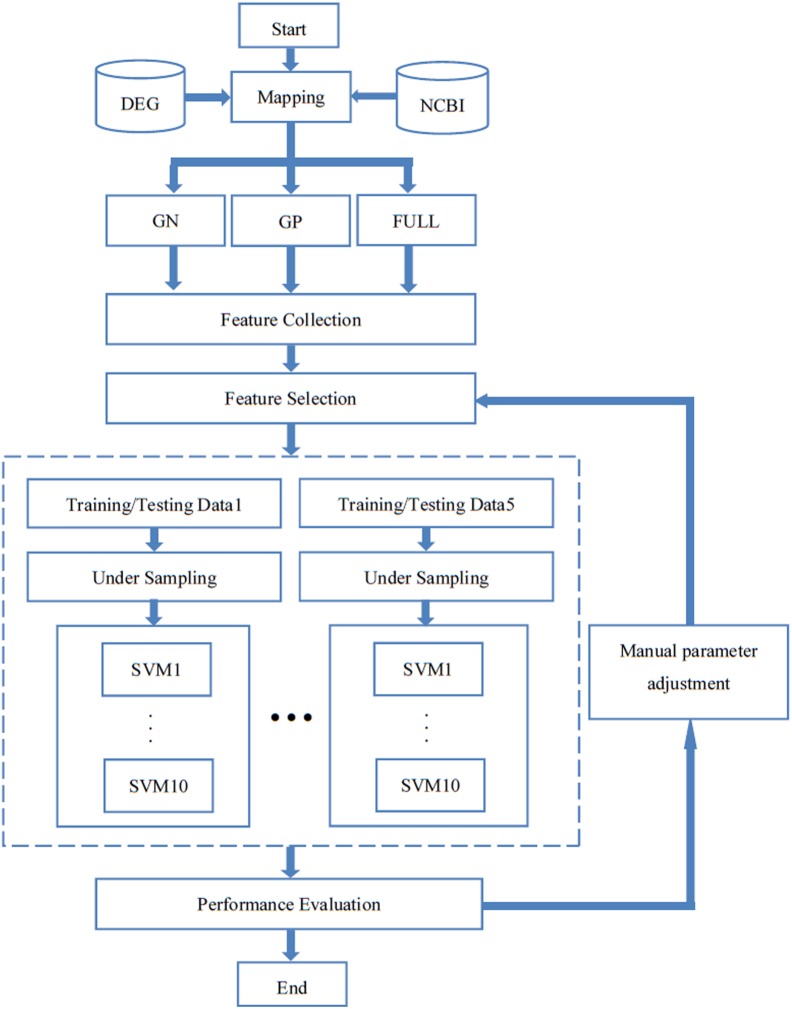
Workflow of analysis procedures.

The workflow is illustrated in Fig 1.

(1) Data was downloaded.

The data downloaded from DEG and GenBank were mapped according to the NCBI gene identifier number. The genes from DEG were labeled as essential, with the remaining genes labeled as nonessential.

(2) Feature collection and selection.

The frequently used sequence-based features were collated and are listed in Table 2. Each attribute was scaled to the [−1, +1] interval, and an initial dataset was obtained. The Lasso contains a penalty term that constrains the size of the estimated coefficients. As the penalty term increases, the Lasso sets more coefficients to zero. In this study, the Lasso was used with 10-fold cross validation. The selected features are marked with an asterisk in Table 2.

(3) Training and prediction.

A SVM is an efficient classification algorithm that is suitable for solving binary classification problems in high-dimensional spaces [25]. LibSVM 3.18 was used to assess the effectiveness of the subset of selected features [26]. Grid optimization was used to determine and optimize the SVM parameters based on the radial basis function kernel for the cross-validation. The difference between the number of essential and nonessential genes was sufficiently large (Table 1) that an under-sampling strategy was also used to deal with the data imbalance [27].

(4) Performance evaluation.

For the GN, GP, and Full datasets, the dimensions of the reduced features were 37, 38, and 40, respectively (Table 2). For each dataset, two-thirds of the total number of gene was assigned as a training set, and the rest was used as a test set. The parameters for SVM were optimized through a 5-fold cross-validation of the training set. The trained and optimized classifier was then evaluated using the test set. This process was repeated five times with different random splitting of the training and test data sets, and the under-sampling of the dataset of nonessential genes was repeated 10 times. Each result represents the averaged value over the 50 repetitions.

**Fig 2:**
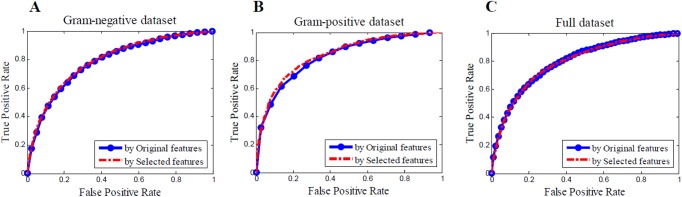
Three ROC curves for predicting essential genes based on the original and selected features. (A) ROC curves for Gram-negative dataset. (B) ROC curves for Gram-positive dataset. (C) ROC curves for Full dataset.

A receiver operator characteristics curve (ROC-curve) was used to measure the classification performance. The area under the ROC-curve (AUC) yields a performance estimate across the entire range of thresholds (Fig 2). The indexes related to prediction accuracy (ACC), AUC, sensitivity, specificity, positive predictive value (PPV, i.e., precision), and the average of specificity and sensitivity (AVE) were also calculated to assess the efficiency (Table 3) [28].

**Table 3:** Comparison of the classification results of original and selected features.

	Gram-Negative			Gram-Positive			Full		
	Original features	Selected features	Variation	Original features	Selected features	Variation	Original features	Selected features	Variation
Sensitivity	0.695	0.713	0.019	0.737	0.729	-0.009	0.708	0.715	0.007
Specificity	0.737	0.733	-0.005	0.752	0.769	0.016	0.743	0.736	-0.006
AVE	0.716	0.723	0.007	0.745	0.749	0.004	0.725	0.726	0.000
AUC	0.782	0.790	0.009	0.826	0.828	0.002	0.797	0.794	-0.003
Number of features	59	37	-22	58	38	-20	57	40	-17
Optimization time (Sec)^a^	56439	46421	-17.750%	3671	3003	-18.197%	140375	116976	-16.669%
Classification and prediction time (Sec)	2245	1175	-47.661%	152	112	-26.316%	3853	3399	-11.783%
Total running time (Sec)	58684	47596	-18.894%	3823	3115	-18.519%	144228	120375	-16.538%

^a ^The optimized parameters include C and gamma, and were determined using the Grid Method with default parameters.

(5) Steps 2–4 were then repeated to obtain the key features.

Based on performance evaluation results from step 4, we estimated the efficiency of the feature selection. If the prediction accuracy decreased, the subset of selected features was assessed as unqualified. Steps 2–4 were repeated with manual parameters adjustment until the accuracy reached the same level as provided by the original features. The features obtained at the end of the analysis were retained as key features.

## Results

The original features which are widely used in existing prediction models and the key features selected by the Lasso are listed in Table 2.

For machine learning methods, as the number of features decreased, the computational complexity also decreased. This was affected by many factors, including the number of genes in the training dataset, the number of dimensions, and the number of support vectors [29,30]. In addition, as the feature dimensions decreased, the computational time also decreased, while the computational efficiency increased.

We know that some sequences in the GN, GP and Full dataset are closely related. As a result, the corresponding genes could be very similar, and there exist redundant in the dataset. Considering overfitting and overestimate of the performance of the algorithm, three evaluation methods were designed to evaluate the effectiveness of the selected features. Firstly, the comparison of the computational performance on the selected features before and after feature selection of three dataset (GN, GP and Full dataset) was carried out. The next one was the performance comparison in 31 diverse species, separately. Finally, leave-one-species out method was employed, i.e., genes of thirty species were used as training set and gens of another species as testing set, in turn.

All analysis was performed on an Intel I7-4790 (3.6 GHz) computer with 16 GB memory and a 64-bit Windows operation system.

### Performance evaluation before and after feature selection in the GN, GP and full dataset

A comparison of the computational performance before and after feature selection for the three datasets is shown in Table 3.

(1) Feature selection and evaluation in the GN dataset

The GN dataset contained 21 bacterial genomes (Table 1), including 7980 essential genes and 73379 non-essential genes. Twenty-two redundant features were eliminated. The AUC, sensitivity, specificity, and AVE are listed in Table 3. Prediction accuracy remained stable after feature selection. The ROC curve showed that the performance was equivalent before and after feature selection (Fig 2). Furthermore, we observed an improvement in program running time when the selected features were used. The parameter optimization time was decreased by 17.8%, the classification and prediction time was decreased by 47.7%, and the total running time was decreased by 18.9%.

(2) Feature selection and evaluation in the GP dataset

The GP dataset contained ten bacterial genomes, including 3019 essential genes and 19246 non-essential genes. Twenty redundant features were eliminated. The parameters of prediction performance are listed in Table 3. For this dataset, the parameter optimization time was reduced by 18.2%, the classification and prediction time was decreased by 26.3%, and the total running time as reduced by 18.5%.

(3) Feature selection and evaluation in the Full dataset

The Full dataset, containing all 31 bacterial species, included 10999 essential genes and 92625 non-essential genes. The common features between Gram-negative and Gram-positive bacteria were collated, and the original dataset contained 57 features. Comparative results of feature selection are listed in Table 2. Using the selected features, the parameter optimization time was decreased by 16.7%, the classification and prediction time was reduced by 11.8%, and the total running time was decreased by 16.5%. In addition, the prediction accuracy remained stable after the removal of 17 redundant features.

(4) Comparison of the prediction performance

Here, we chose eight representative papers and compared the prediction performance. The methods used in these papers were mainly based on machine learning. The commonly used performance indexes in these papers were chosen and listed in Table 4 for comparison. In this study, 31 bacterial objects (21 Gram-negative objects and 10 Gram-positive objects) were analyzed, more than the objects analyzed in the other studies.

**Table 4:** Comparison of the prediction performance.

	Our Results			Saha S, *et al*. [7]		Song K, *et al*. [28]			Ning LW, *et al*. [31]^5^			Deng J, *et al*. [12]	Gustafson AM, *et al*. [9]	Gerdes SY, *et al*. [32]	Joyce AR, *et al*. [33]	Plaimas, *et al*.[11]	Ye YN, *et al*. [34]	
	GN	GP	FULL (GN+GP)	*S*. *cerevisiae*		*E*. *coli*^2^	*B*. *subtilis*^3^	Max	*E*. *coli*	*M*. *pulmonis*	Combine	*E*. *coli*	*E*. *coli*	*E*. *coli*	*E*. *coli*	*E*. *coli*	*E*. *coli*	
				SVM	KNN^1^			(Min)^4^									BLAST^6^	CEG_MATCH^6^
Sensitivity	0.709	0.733	0.715	0.768	0.742	0.760	0.792	0.904	/	/	/	0.73	0.52	0.68	0.26	/	/	/
								(0.609)										
Specificity	0.733	0.786	0.736	/	/	0.867	0.858	0.926	/	/	/	0.92	0.96.	0.88	0.25	/	0.431	0.60
								(0.778)									(0.345)	(0.694)
AVE	0.721	0.760	0.726	/	/	0.814	0.825	0.898	/	/	/	/	/	/	/	/	/	/
								(0.735)										
AUC	0.789	0.838	0.794	0.81	0.81	0.866	0.870	0.937	0.82	0.74	0.76	/	/	/	/	0.81	/	/
								(0.804)								(0.75)		
ACC	0.731	0.763	0.734	0.741	0.734	0.904	0.903	0.960	/	/	/	/	/	/	/	/	0.694	0.712
								(0.813)									(0.677)	(0.701)
PPV	0.226	0.330	0.243	0.731	0.730	0.709	0.673	0.942	/	/	/	0.44	0.53	0.33	0.42	/	/	/
								(0.435)										
Number of feature	37	38	40	13	13	494^7^	494^7^	/	158	158	158	13	28	/	/	33^8^	/	/
Number of object	21	10	31	1	1	11	11	/	1	1	16	1	1	1	1	3	16^9^	16^9^

^1 ^k-nearest neighbor (KNN) method

^2^ Date of *E*. *coli* was used for training. The data of the other 11 objects were used as test set, and the results were averaged.

^3^ Date of *B*. *subtilis* was used for training. The data of the other 11 objects were used as test set, and the results were averaged.

^4 ^The maximum and the minimum values of the prediction results.

^5^ Results based on cross validation were chosen for comparison.

^6^ BLAST: Identity >50. CEG_MATCH: K = 3.

^7 ^The features were the 93’ Z-curve features (252 variables), orthologs values (187), and other DNA or amino acid sequence based features (55).

^8^ The features were the topology features (25) and the genomic and transcriptomic features (8).

^9^ Results of 16 objects were listed in [34].

A remarkable performance was provided in [28]. It should be noticed that the features used in the work listed in Table 4 were mainly sequence based feature (see note 7 and 8 of Table 4). The features used in [28] included the 93’ Z-curve features, orthologs, and other DNA or amino acid sequence based features. The Z-curve features provided more topology information, which was an important factor to get higher prediction accuracy.

A basic method for prediction of essentiality of genes is based on homology with essential genes experimentally determined in other bacterial species. So we compared the results of our method with the prediction results of BLAT and a homology alignment based method (CEG_MATCH), which are shown in the last two columns of Table 4.

In general, results show our method provides higher accuracy and specificity. And the other comparative results were provided by machine learning based methods. The prediction performance of our method provided the same accuracy level. It showed the generalization ability of our method and the efficiency of the screened features.

### Performance evaluation in 31 diverse species

A comparison of prediction of essential gene within and between species before and after feature selection for 31 diverse species was shown in S1 Table. To facilitate the analysis, the 40 selected features from Full dataset were used in the prediction.

(1) All the genes of a species were used for training the predicting model, and then were predicted by the model, in self-test method. Of course, an excellent prediction result was obtained.

(2) In a 5-fold cross-validation method, four-fifth of the total genes of a species was assigned as a training set, and the rest of this species was used as a test set. This process was repeated five times for each prediction.

(3) In a pairwise method, the total number of gene of one species was assigned as a training set, and the genes of every other species were used as a test set.

In total, the prediction accuracy remained stable after the removal of 17 redundant features. The average AUC got a slight increase and the running time was decreased.

### Performance evaluation based on leave-one-species out method

The genes of thirty species were used as training set and genes of another species as testing set, in turn. Again, the 40 selected features from Full dataset were used in the prediction, to facilitate the analysis. (See S2 Table for detail.)

A SVM classifier was constructed based on the corresponding training set, and used to predict the genes of the left specie. This process repeated 31 times. A comparison of the prediction performance between before and after feature selection was shown in S2 Table. The prediction accuracy remained stable after the removal of 17 redundant features. The average AUC got a slight decrease (0.30%) and the running time was decreased.

## Discussion

Essential genes are absolutely necessary for the survival of an organism [2]. Investigating features associated with gene essentiality is fundamental to the prediction and identification of essential genes. Machine learning methods are widely used in this field, and numerous features have been proposed and employed to improve calculation accuracy. It is important to understand the relationship between features and gene essentiality, and thus to identify the key features.

In this study, Lasso was applied for feature selection to predict essential genes from 31 bacterial species. For the GN, GP, and Full datasets, the feature dimensions were decreased from 59, 58, and 57, to 37, 38, and 40, respectively. To assess the effect of these reductions, corresponding SVM classifiers were built based on original and selected features, and then used to predict the essential genes of the selected bacteria. As some closely related genes may lead to overfitting and overestimate on the performance of the algorithm, three evaluation methods were designed to evaluate the effectiveness of the selected features more comprehensively. In all cases, the prediction accuracy remained stable after the feature set was reduced. The results showed that there was a high degree of redundancy amongst the features for predicting gene essentiality. The resulting subset of features will be of use for further research, including gene prediction, synthetic biology, and drug design studies. Furthermore, the closely related genes may also affect the feature selection and result in feature bias, which should be considered for further study.

It should be noted that structural and functional features, such as protein-protein interactions and gene expression, which cannot be directly derived from the sequence, were not considered in this study. In practice, only sequence-based features are commonly available for a newly sequenced genome. Amongst the 31 bacterial genomes examined in this study, very few had corresponding structural or functional data available. As the aim of computational prediction methods is to reduce or even eliminate the dependency on biochemical experiments, just like that the long-range goal of Protein Structure Initiative (PSI) is to make the atomic-level structures of most proteins easily obtainable from their corresponding DNA sequences [35], we focused on sequence-based features for application in biological research.

However, our results indicated that the prediction accuracy could still be improved (Table 3). Although Table 4 shows the generalization ability of our method and the efficiency of the screened features, Table 3 shows the GP gets relatively better prediction performance than GN gets (Sensitivity, Specificity, AVE, and AUC). We suppose that is because the GN contains 21 objects, much more than the GP (10 objects). This encourages us promote the generalization ability and the efficiency of our method further, to provide the same performance for different objects. We hope to address this by pursuing new features that are closely related to gene essentiality, and by improving and optimizing the classifying algorithm. Furthermore, the development and improvement of comprehensive and specific databases for essential genes, such as the Database of Essential Genes (DEG), will provide a greater number of samples, which will enhance our prediction model. As described by Viktor and Kenneth, a simple algorithm based on a large dataset will provide better results than a comprehensive algorithm based on limited data [36].

Finally, we observed that the program running time (including training, optimizing, and testing) decreased after feature selection. With the rapid progress of computational ability, researchers can pay more attention to computation accuracy, instead of computation efficiency. However, with increasingly large datasets, greater computation efficiency is always advantageous.

## Conclusion

Feature selection is a critical factor in classification task. Inclusion of too many features affects the generalization ability and increases computational complexity. Therefore, it is desirable to use as few features as possible to carry out a classification, while maintaining the same level of accuracy. In this study, we screened the key biological features related to gene essentiality using Lasso, eliminated the redundant features, and assessed the validity of our selection in 31 bacterial species. The results could be of use for further research projects, including *in silico* gene prediction, synthetic biology, and drug design studies.

## Supporting information

S1 File Information of the 31 analyzed objects. (RAR)

S1 Table Comparison of the classification results of original and selected features in 31 diverse species. (XLSX)

S2 Table Comparison of the classification results of original and selected features. (XLSX)
